# Vital Sign and Biochemical Data Collection Using Non-contact Photoplethysmography and the Comestai Mobile Health App: Protocol for an Observational Study

**DOI:** 10.2196/65229

**Published:** 2025-04-28

**Authors:** Gianvincenzo Zuccotti, Paolo Osvaldo Agnelli, Lucia Labati, Erika Cordaro, Davide Braghieri, Simone Balconi, Marco Xodo, Fabrizio Losurdo, Cesare Celeste Federico Berra, Roberto Franco Enrico Pedretti, Paolo Fiorina, Sergio Maria De Pasquale, Valeria Calcaterra

**Affiliations:** 1 Department of Biomedical and Clinical Science University of Milano Milano Italy; 2 Pediatric Department Buzzi Children’s Hospital Milano Italy; 3 Come Stai S.p.A Milano Italy; 4 Endocrine Diseases and Diabetology Unit Azienda Socio Sanitaria Territoriale Fatebenefratelli Sacco Milano Italy; 5 Diabetology and Endocrinology Unit Istituto di Ricovero e Cura a Carattere Scientifico MultiMedica Milano Italy; 6 Cardiac Rehabilitation Unit Istituto di Ricovero e Cura a Carattere Scientifico MultiMedica Milano Italy; 7 International Center for Type 1 Diabetes, Pediatric Clinical Research Center Romeo and Enrica Invernizzi University of Milano Milano Italy; 8 Pediatric and Adolescent Unit, Department of Internal Medicine University of Pavia Pavia Italy

**Keywords:** biochemical data, mHealth, mobile app, non-contact photoplethysmography, detection, Comestai, data accuracy, monitoring, vital sign measurement, screening

## Abstract

**Background:**

Early detection of vital sign changes is key to recognizing patient deterioration promptly, enabling timely interventions and potentially preventing adverse outcomes.

**Objective:**

In this study, vital parameters (heart rate, respiratory rate, oxygen saturation, and blood pressure) will be measured using the Comestai app to confirm the accuracy of photoplethysmography methods compared to standard clinical practice devices, analyzing a large and diverse population. In addition, the app will facilitate big data collection to enhance the algorithm’s performance in measuring hemoglobin, glycated hemoglobin, and total cholesterol.

**Methods:**

A total of 3000 participants will be consecutively enrolled to achieve the objectives of this study. In all patients, personal data, medical condition, and treatment overview will be recorded. The “by face” method for remote photoplethysmography vital sign data collection involves recording participants’ faces using the front camera of a mobile device (iOS or Android) for approximately 1.5 minutes. Simultaneously, vital signs will be continuously collected for about 1.5 minutes using the reference devices alongside data collected via the Comestai app; biochemical results will also be recorded. The accuracy of the app measurements compared to the reference devices and standard tests will be assessed for all parameters. CIs will be calculated using the bootstrap method. The proposed approach’s effectiveness will be evaluated using various quality criteria, including the mean error, SD, mean absolute error, root mean square error, and mean absolute percentage error. The correlation between measurements obtained using the app and reference devices and standard tests will be evaluated using the Pearson correlation coefficient. Agreement between pairs of measurements (app vs reference devices and standard tests) will be represented using Bland-Altman plots. Sensitivity, specificity, positive predictive value, negative predictive value, accuracy, and likelihood ratios will be calculated to determine the ability of the new app to accurately measure vital signs.

**Results:**

Data collection began in June 2024. As of March 25, 2025, we have recruited 1200 participants. The outcomes of the study are expected at the end of 2025. The analysis plan involves verifying and validating the parameters collected from mobile devices via the app, reference devices, and prescheduled blood tests, along with patient demographic data.

**Conclusions:**

Our study will enhance and support the accuracy of data on vital sign detection through PPG, also introducing measurements of biochemical risk indicators. The evaluation of a large population will allow for continuous improvement in the performance and accuracy of artificial intelligence algorithms, reducing errors. Expanding research on mobile health solutions like Comestai can support preventive care by validating their effectiveness as screening tools and guiding future health care technology developments.

**Trial Registration:**

ClinicalTrials.gov NCT06427564; https://clinicaltrials.gov/study/NCT06427564

## Introduction

In recent decades, there has been an increasing focus on self-monitoring apps in primary care, which, with the advent of new technologies, have become more convenient and accessible for patients [1-3].

The use of mobile health (mHealth) apps is undeniably a valuable tool for enabling self-monitoring and health care interventions [4,5]. Specifically, the advancement of noncontact techniques for monitoring human vital signs holds significant potential to enhance patient care across various settings [6,7].

By enabling easier and more convenient monitoring, these techniques can mitigate serious health issues and improve patient outcomes, particularly benefiting individuals who cannot or prefer not to visit traditional health care settings. Vital signs are objective measures of the essential physiological functions of a living organism and they are the critical starting steps in any clinical evaluation.

Early detection of changes in vital signs typically correlates with faster identification of changes in the patient’s health status and the escalation of care if necessary [8]. Alterations in vital signs preceding clinical deterioration are well documented, and the early identification of preventable outcomes is crucial for timely intervention [8]. Logically, the more frequently vital signs are measured, the quicker clinical deterioration can be detected.

The app named Comestai is a noninvasive and easy-to-use tool that enables the measurement of different vital parameters. The app allows users to simultaneously measure vital parameters such as heart rate (HR), respiratory rate (RR), oxygen saturation (SpO_2_), blood pressure (BP), hemoglobin (Hb), and glycated hemoglobin A_1c_ (HbA_1c_) using the photoplethysmographic method with the front camera of a mobile device.

Photoplethysmography is a practical method for noncontact vital sign measurement using a red-green-blue (RGB) camera that has been recently introduced [6,9]. Photoplethysmography uses a transducer that emits infrared light from an LED into the dermis. The adjacent photodetector measures the backscattered light, which is then represented as a line tracing. The intensity of the backscattered light changes with the volume of red blood cells in the dermal capillaries [9]. Photoplethysmography captures color change signals collected from various regions of the face, correlating the estimated magnitudes of red cell aggregation influenced by blood perfusion with the reflected light intensity in each region. By comparing variations in the averaged color signals, the camera-based signal-to-noise ratio estimates are enhanced.

As reported by [10,11], photoplethysmography has shown promising results in measuring vital signs and could be a valuable tool for the early detection and screening of cardiovascular, respiratory, and metabolic conditions. These interventions could be crucial in combating noncommunicable chronic diseases (NCDs) [12]. Furthermore, literature data on the use of photoplethysmography for vital sign detection usually refer to controlled clinical settings. Larger, more diverse populations need to be included to ensure accuracy across various demographics and health conditions [10,12,13].

In this study, vital parameters will be measured using the Comestai app to confirm the accuracy of photoplethysmography methods compared to standard clinical practice devices, analyzing a large and diverse population. In addition, the app will facilitate big data collection to enhance the algorithm's performance in measuring Hb, HbA_1c_, and total cholesterol. The results will be useful to determine whether this user-friendly mobile app achieves adequate accuracy and can be adopted to enhance self-monitoring of health status and detect early health deterioration. A secondary result will be the collection of raw data that can be used to train the algorithm to detect other vitals.

## Methods

### Recruitment

The objectives of this study will be achieved by recruiting 3000 participants from the outpatient clinic of ASST-Fatebenefratelli-Sacco, Buzzi Children’s Hospital, and IRCCS MultiMedica in Milan, Italy. All participants who meet the inclusion criteria and provide consent to participate will be enrolled consecutively. Recruitment will take place from June 2024 to September 2025.

### Eligibility Criteria

Participants eligible for inclusion in the study must meet the criteria in Textbox 1.

In addition to the above criteria, 70% of the target population should include adult participants meeting at least one of the conditions in Textbox 2. Family history of diabetes or hypertension will also be investigated.

The procedures and evaluations in Textbox 3 will be performed during screening to enroll eligible participants.

Characterizing aspects will be noted, such as scars, piercings, beards, glasses, or the use of facial products, sunscreen cream, or nail polish. Furthermore, current pregnancy will be reported.

**Figure 1:**
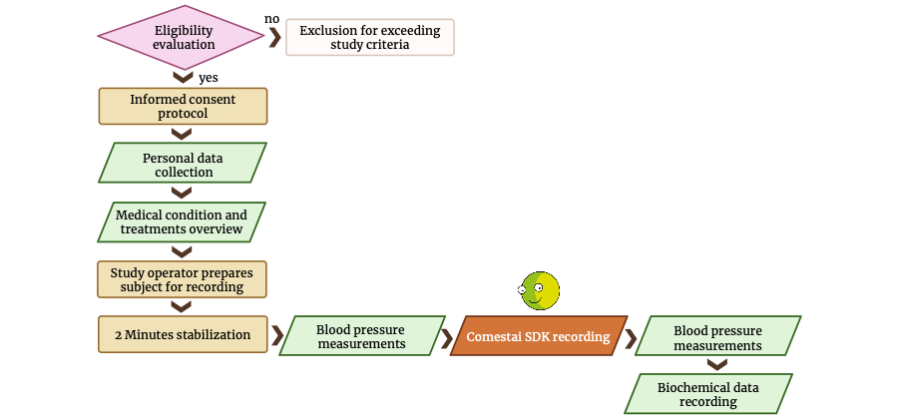
Process flowchart.

The flowchart of the process is schematized in Figure 1.

**Inclusion and exclusion criteria.**

**Inclusion criteria**

- Must demonstrate the ability to comprehend and provide written informed consent.
- Male or female participants aged ≥16 and ≤65 years.
- Must be willing and able to adhere to study procedures.

**Exclusion criteria**

Participants will be excluded if they meet any of the following conditions:

- Compromised circulation, injury, or physical abnormalities affecting fingers, hands, ears, forehead/skull, or other regions of interest essential for study testing.
- The presence of tattoos in the optical path hinders the assessment of necessary regions of interest.
- Severe allergies to standard adhesives, latex, or other materials used in medical sensors for measuring vital signs.
- Medical conditions that, in the investigator’s opinion, prevent the conduct of study assessments.
- Considered unfit for participation by the investigator.

**Additional inclusion criteria.**

1. Prediabetes or diabetes with glycated hemoglobin (HbA_1c_) levels between 5.7% and 13%:
  - 30% of participants with HbA_1c_ 5.7%-6.4%.
  - 30% of participants with HbA_1c_ >6.4%.
2. Hypertension with systolic measurements exceeding 130 mm Hg:
  - 40% of participants with systolic blood pressure >130 mm Hg.
  - 20% of participants with systolic blood pressure >160 mm Hg.
3. Total cholesterol:
  - 40% of participants with levels >200 mg/dL and/or low-density lipoprotein cholesterol >130 mg/dL.
4. Atrial fibrillation (~1% prevalence).
5. Smokers, constituting approximately 20%-30% of the study cohort.

**Procedures and evaluations.**

- Obtain participant consent and, if agreed, additional consent for a blood sample.
- Review eligibility: assess eligibility as described in the inclusion and exclusion criteria.
- Record the participants’ medical and medication history: each medical problem encountered is classified into the sector of the body involved and for each of these, the starting date and the drugs used for treatment are indicated.
- Record participant demographics, including date of birth, age, smoking, and alcohol drinking habits.
- Record body characteristics including height, weight (BMI will be calculated as a person’s weight in kilograms divided by the square of height in meters), and skin tone using the Fitzpatrick scale
- Interview the patient about his feelings in the last 2 weeks and if he has suffered from problems such as anxiety, nervousness, lack of control, loss of interest in doing things, or depression.

### Technology

Comestai is a mobile app that leverages a combination of signal processing and artificial intelligence (AI) technologies, along with a proprietary mathematical backend server, to analyze the skin of the upper cheek region through a smartphone’s front camera (iOS or Android).

**Figure 2:**
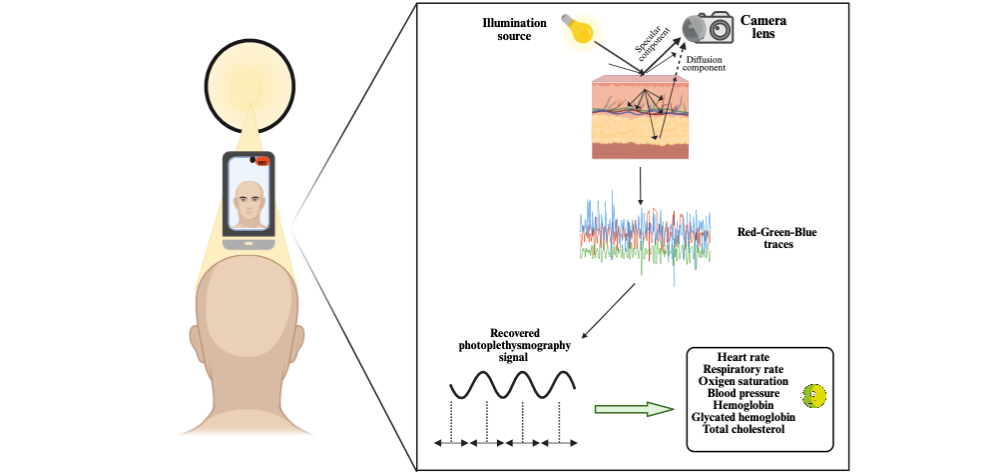
Remote photoplethysmography from facial videos. RGB: red-green-blue.

The Comestai app captures video images using signal processing AI technologies and analyzes the data using photoplethysmography techniques (Figure 2) [9,14-16]. This approach provides real-time measurements of physiological parameters, including SpO_2_, HR, PR, and RR, and biochemical parameters Hb, HbA_1c_, and total cholesterol. Unlike other apps, the app uses a novel technology that extracts ambient light RGB signals from face videos in real time, eliminating the need for cloud servers or an internet connection.

### Equipment for Data Collection

For vital signs data collection using the Comestai app, the study staff will use the following mobile phones for remote photoplethysmography “by face” measurements:

1. Face—iPhone 13 Pro (10 bit) and Samsung Galaxy S21 Ultra
2. A total of 2 additional phones will be used remotely—Android phone (recorder remote for initiating measurements from a distance) and iPhone (Masimo app for collecting data from the Masimo finger sensor).
3. Other necessary equipment includes phone stands (2 per measurement room).

In addition to the equipment for vital signs data collection using the Comestai app, reference devices will be used to measure the participant’s vital signs for comparative purposes.

#### Vital Sign Data Collection Using the Comestai App and Reference Devices

The study personnel will gather vital signs data in a designated room equipped with the following amenities: a table, a chair with a headrest and arm support, a white wall for participants to sit against, stable Wi-Fi, power sockets, and adequate lighting (if the room lacks sufficient illumination, one Theralite lamp per room will be necessary).

Vital signs data will be gathered using the Comestai app through the “by face” method and with the reference devices detailed in Table 1.

**Table 1:** Reference devices for measuring vital signs.

Device	Saturation of oxygen	Pulse rate	Respiratory rate	Blood pressure
Withings BPM Connect				✓
Masimo mightySat Rx	✓	✓	✓	
Polar Verity Sense		✓		

**Figure 3:**
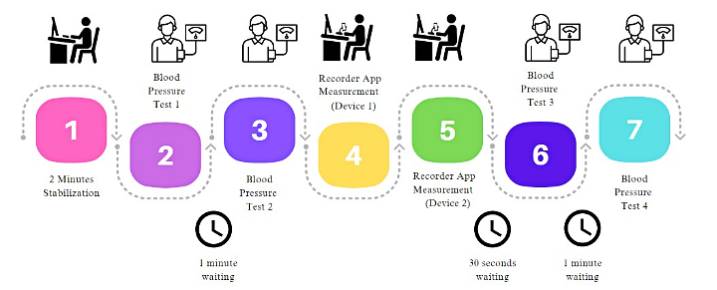
Measurement procedure flow.

Specifically, as reported in Figure 3, the measurement procedure flow will be as follows: first, The participant should sit for 2 minutes for stabilization with references connected. Second, baseline distance will be set at approximately 25 cm (closest as possible). Third, we will measure BP twice (if systolic ±10 and/or diastolic ±5, we will make a third measurement), waiting 1 minute in between the 2. Fourth, we will make 1 recording with each device. Fifth, we will measure BP twice (if systolic ±10 and/or diastolic ±5 make a third measurement).

The “by face” method for remote photoplethysmography vital signs data collection involves recording participants’ faces using the front camera of mobile devices (iOS and Android) for approximately 1.5 minutes.

During “by face” data collection, participants will be seated comfortably, facing a screen at approximately 25 cm, with their faces fully visible (without wearing masks, hats, or any facial accessories). Participants are required to maintain stable breathing and minimal movement and to gaze directly at the camera throughout the data collection process.

Simultaneously, vital signs data (SpO_2_, RR, BP, and PR) will be continuously collected for about 1.5 minutes using the reference devices alongside data collected via the Comestai app.

BP measurements will be taken using the reference devices before and immediately after data collection with the Comestai app.

### Data Collection and Management

Data from the investigational device will be collected by trained staff members. Vital signs data obtained using the app and reference data will be stored on a secured cloud. The app will be activated only when the cell phone is offline, ensuring that no information is stored in the Comestai cloud. Consequently, the company will not have access to any data collected during the tests.

In addition, the app will be deleted after use to prevent any data from being uploaded into the system. Data collected using the reference devices will be stored without any identifiable participant information and will only be identifiable using the participant identification number provided during screening.

The raw data of participants’ vital signs will be locked, encrypted, and accessible only to the study center team. Raw data can be processed only after unlocking the files to calculate the final measured results.

The coordinator center (Buzzi Children’s Hospital) will be responsible for securely storing the data in a locked and secured location. The Endocrinology Unit of ASST-Fatebenefratelli-Sacco and Buzzi Children’s Hospital will handle data entry and ensure data quality.

This study will be conducted in compliance with the Good Clinical Practice guidelines, including the archiving of essential documents.

No medical decisions will be made based on the data obtained from the Comestai app, reference devices, or blood sample results.

### Clinical Data Cleaning and Validation

Clinical data for the study will be meticulously organized, easily accessible, and thoroughly cleaned both throughout and after the study concludes (post data lock). Activities in clinical data management encompass data collection and entry, tracking electronic case report form (eCRF) usage, database design, data validation, and database locking.

As previously outlined, the eCRF will serve as the primary tool for capturing clinical data. The data fields within the eCRF are clearly defined and maintain consistency throughout. The eCRF is designed to be succinct, intuitive, and user-friendly.

Database edit checks will be conducted to identify discrepancies in the data, which could stem from inconsistent entries, missing data points, range discrepancies, or deviations from the study protocol. Any incomplete discrepancies will be addressed by site investigators. Continuous quality control measures for data processing will be implemented at regular intervals throughout the study duration.

### Criteria for Data Collection and Study Interruption

Temporal interruption of vital signs data collection using the app may occur in the following cases:

- Technical issues with the investigational and/or reference devices
- The study may be interrupted for the following reasons:
- Breach of participant confidentiality.
- Decision by the principal investigator or institutional ethics committee.
- The investigators may also decide to terminate the study early if sufficient data has been collected for algorithm improvement.

### Statistical Analysis

Numerical variables will be presented as minimum and maximum values, means, SDs, medians, and quartiles. Categorical variables will be presented as absolute and relative frequencies.

The accuracy of the app measurements compared to the reference devices and standard tests will be assessed for all parameters. CIs will be calculated using the bootstrap method. Participants with abnormal adverse events (defined as 3 SDs or more above the mean) and those with invalid reference device values will be excluded from the analysis.

In order to provide a more comprehensive view of the model’s performance, the proposed approach’s effectiveness was evaluated using various quality criteria, including the mean error, SD, mean absolute error, root mean square error, and mean absolute percentage error. The correlation between measurements obtained using the app and those from reference devices and standard tests will be evaluated using the Pearson correlation coefficient. The agreement between the 2 sets of measurements (app vs reference devices/standard tests) will be represented using Bland-Altman plots. Sensitivity, specificity, positive predictive value, negative predictive value, accuracy, and likelihood ratios will be calculated to determine the ability of the new app to accurately measure vital signs.

### Sample Size Estimation

Considering the average values of pressure/saturation/HR among a general population with a sample of 3000 participants, there will be a power exceeding 95% for all evaluated parameters in a 2-sided test with a *P* value of .05 if the discrepancy between the 2 measurement methods is as reported in Table 2.

**Table 2:** Expected error level of the measurements.

Vital	Range	Unit of measurement	Error level
Pulse rate	50-180	BPM^a^	RMSE^b^ ≤3
Respiratory rate	8-30	BrPM^c^	RMSE ≤3
Blood pressure systolic	80-180	mm Hg	MAE^d^ ≤15
Blood pressure diastolic	50-120	mm Hg	MAE ≤10

^a^BPM: beats per minute.

^b^RMSE: root mean square error.

^c^BrPM: breaths per minute.

^d^MAE: mean absolute error.

### Known Potential Risks and Benefits of the Investigational Device

#### Potential Risks

No adverse device effects are expected from the Comestai app, and there are no contraindications for its use in the study population. However, unforeseeable risks related to the data collection process may arise.

One such risk is the potential breach of participant confidentiality due to improper data storage. In order to mitigate this, vital signs data will be securely stored on Comestai’s cloud. Videos of faces obtained using the “by face” method will be anonymized, and recognized only by the participant identification number assigned during screening. The raw vital signs data will be locked, encrypted, and accessible solely by the study center team. Processing of this data will only occur once the files are unlocked.

#### Potential Benefits

Participants in this study will not receive direct benefits. However, the data gathered may aid in the advancement of the Comestai noninvasive software app for monitoring vital signs. Progress in this area has the potential to enhance patient care quality and reduce labor time and costs within the health care system.

Advances in this field may improve the quality of care provided to patients and save labor time and costs for the health care system.

### Ethics Approval

The study was approved by the ethics committee (Comitato Etico Territoriale Lombardia 1, Milano, Italy, approval code CET 98-2024). The study will be conducted according to the Declaration of Helsinki guidelines, international guidelines, and current clinical trial laws.

All participants will give their written consent after being fully informed about the study. For individuals younger than 18 years, both legal guardians’ written consent and assent by adolescents themselves will be collected.

Data will be collected and stored securely to maintain confidentiality, with only authorized personnel having access to identifiable information.

The study protocol was registered in ClinicalTrials.gov (NCT06427564). If modifications in the original protocol are required, additional amendments will be demanded by the Ethics Committee. In this scenario, the approved related information will be updated on ClinicalTrials.gov.

The authors followed the SPIRIT (Standard Protocol Items: Recommendations for Interventional Trials) guidelines for study protocol reporting (Multimedia Appendix 1) to ensure greater scientific rigor. Written informed consent will be obtained from all participants, and data collection will be performed by a trained research team.

Consent will be requested for the use of the blood sample results (complete blood count, HbA_1c_, lipid profile, glucose, and creatinine). Consenting to view the biochemical data is optional. A participant who chooses not to provide their biochemical results will still be eligible to participate and complete the recordings.

## Results

Data collection began in June 2024. As of March 25, 2025, we have recruited 1200 participants. The outcomes of the study are expected at the end of 2025. The analysis plan involves verifying and validating the parameters collected from mobile devices via the app, reference devices, and prescheduled blood tests, along with patient demographic data.

## Discussion

### Principal Findings

The project aims to report whether this user-friendly mobile app supports a high level of accuracy in monitoring SpO_2_, RR, BP, and PR, demonstrating the same reliability in detecting Hb, HbA_1c_, and total cholesterol. Enhancing algorithm performance and achieving a high level of accuracy will be crucial in assessing whether the app can be adopted to improve self-monitoring of health status. This, in turn, could enhance the screening for early abnormalities in vital signs and biochemical parameters.

### Strengths and Limitations

Today’s health care industry embraces technological innovations to provide better personalized health care services to the public [17]. Mobile-based solutions and apps are one such revolutionary and trending innovation that facilitates enhanced patient care management and efficient diagnosis. They are increasingly reliant on wireless technologies as they evolve [17]. Applications of mHealth typically include the use of mobile devices in the collection of health and clinical data, the provision of health information to practitioners, researchers, and patients, the real-time monitoring of patients; vital signs, and the direct delivery of care (via mobile telemedicine).

In an mHealth context, the advancement of noncontact methods to monitor human vital signs holds substantial promise for enhancing patient care across various environments [6].

A recently proposed RGB camera–based method for noncontact vital sign measurement using photoplethysmography has been identified as an interesting tool for recording vital signs. Photoplethysmography is also used by the Comestai app to assess various vital functions, including SpO_2_, RR, BP, and PR [10]. Initial research into photoplethysmography shows promising outcomes; however, scientific evidence regarding the accuracy and benefits of digital health apps that measure vital signs remains limited and somewhat controversial. Many photoplethysmography studies are still susceptible to errors [18-20], with the majority involving only small sample sizes.

In our protocol, we will recruit a large population (3000 participants) to ensure accuracy across various demographics and health conditions, including also pathological conditions. The use of a large population will also help reduce errors in the results, improving the reliability of the tool. Thorough analysis and classification of these signals are essential for the development of affordable and user-friendly PPG-based tools across a wide range of applications [10].

While there is an increasing number of articles and research on SpO_2_, RR, BP, and PR monitoring using PPG, a limited number of available publications on advancements related to biochemical parameter estimation are reported [10]. Comestai offers the possibility to monitor Hb, HbA_1c,_ and cholesterol concurrently with other parameters, thereby broadening its application possibilities in health care services.

Although the protocol included participants with diseases such as hypertension, prediabetes and diabetes, dyslipidemia, and atrial fibrillation to assess the tool’s accuracy even in the presence of pathological values, conducting the study in a controlled setting can be considered a limitation. Its application in real-life scenarios may present challenges that were not accounted for.

### Future Research

As suggested by the World Health Organization (WHO), it is important to disseminate medical procedures and health programs based on mHealth [21]. Mobile apps have become increasingly popular among professionals and individuals involved in the health care sector, with a variety of mHealth apps developed to improve health care facilities for all kinds of patients [22,23]

Specifically, mobile apps can play a vital role in screening, leading to the early detection of vital sign alterations, which is useful for alerting individuals and their caregivers to access medical care services and design and implement treatment plans. Screening is considered a crucial player in addressing chronic noncommunicable diseases (NCDs), which are responsible for nearly 70% of all deaths worldwide [12,24,25].

According to the WHO [12], NCDs cause 41 million deaths each year. This burden is expected to grow over the next decade, with NCD deaths projected to reach 52 million by 2030. Cardiovascular diseases are the leading cause of NCD deaths, accounting for 17.9 million fatalities annually. They are followed by chronic respiratory diseases (4.1 million) and diabetes (2.0 million, including kidney disease deaths linked to diabetes). Early detection and screening are vital strategies in combating NCDs. Disease screening detects diseases early, often before symptoms appear, through tests, examinations, or other rapid procedures [26]. This early detection enables effective prevention or treatment of identified diseases through timely medical intervention for those at risk [27].

Despite screening being recognized as the gateway to care, routine NCD screening at community and health facilities remains challenging due to limited capacity within health systems. A screening tool must be simple, valid, reliable, quick to administer, cost-effective, and user-friendly [23]. According to reports, photoplethysmography wearables have significant potential for estimating the risk of cardiovascular diseases [28], predicting suspected sleep apnea [29,30], and helping in the clinical diagnosis of hypertension [31] and diabetes. This potential can be harnessed through app solutions.

Recent reviews have assessed the role of apps in aiding self-management and transition among chronically ill young people, enhancing the lifestyles of chronic patients, and improving lifestyle in NCDs [32]. As reported by Moses et al [27], regardless of a country’s economic status, there has been a rise in mobile cellular subscriptions per hundred people in developing nations, mobile app solutions may provide equitable health care solutions without barriers.

### Conclusion

Our study will enhance and support the accuracy of data on vital sign detection through PPG, also introducing measurements of biochemical risk indicators. The evaluation of a large population will allow for continuous improvement in the performance and accuracy of AI algorithms, reducing errors. Expanding research opportunities to evaluate mHealth solutions as effective and dependable screening tools is useful for preventive strategies, reducing the burden of NCDs [27]. App solutions, such as Comestai, may present new opportunities and challenges related to technology use in the health care sector, offering insights and guiding future research directions.

## Appendix

Multimedia Appendix 1 SPIRIT checklist.

## Abbreviations

AI: artificial intelligence
BP: blood pressure
eCRF: electronic case report form
HbA_1c_: glycated hemoglobin
HR: heart rate
mHealth: mobile health
NCD: noncommunicable disease
RGB: red-green-blue
RR: respiratory rate
SPIRIT: Standard Protocol Items: Recommendations for Interventional Trials
SpO_2_: oxygen saturation
WHO: World Health Organization

## Data Availability

The data that will support the findings of this study will be derived from participants’ record data. All data generated or analyzed during this study will be included in the published article and its supplementary information files.
